# Effects of scleral cross-linking using genipin on the process of form-deprivation myopia in the guinea pig: a randomized controlled experimental study

**DOI:** 10.1186/s12886-015-0086-z

**Published:** 2015-07-29

**Authors:** Mengmeng Wang, Christine Carole C. Corpuz

**Affiliations:** Hebei Provincial Eye Hospital, Hebei Provincial Ophthalmology Key Lab, No.399 Quanbeidong Street, Xingtai City, Hebei Province 054001 China; Eye Can Philippines, Inc., San Juan City, Metro Manila Philippines

**Keywords:** Sclera, Cross-linking, Genipin, Form-deprivation, Myopia, Guinea pig

## Abstract

**Background:**

Scleral cross-linking (CXL) is a novel attempt to slow down the axial elongation process in animal eyes. As a natural CXL reagent, genipin would be also effective for the prevention of myopia process. Thus, the present study was designed to evaluate the effects of scleral cross-linking using genipin on the form-deprivation (FD) myopia process of guinea pigs.

**Methods:**

Twenty-seven 3-week-old pigmented guinea pigs were randomly divided into three groups. Group A (*n* = 8) is the untreated control group. Group B (*n* = 8) is the FD control group, where all eyes were induced with monocular FD for 21 days. In Group C (*n* = 11), a sub-Tenon injection of 0.10 mL 0.50 % genipin was performed on FD eyes at day 0, 7 and 14 during the 21-day monocular FD. The ocular refraction, axial length, biomechanical test and light and electron microscopy were measured on all eyes to check the efficacy and safety of this scleral CXL technique.

**Results:**

Compared with Group A, significant increases in myopic refractive errors, axial elongation and reductions of scleral fibril diameter and density were observed in the 21-day FD eyes of Group B (*P* < 0.05). In Group C, the scleral CXL resulted in less myopia and axial elongation as compared with Group B (*P* < 0.05); a significant thickening of scleral fibrils was found after sub-Tenon injections of genipin; no histological damage on the retina or choroid was observed in Group C at the end of this study.

**Conclusions:**

The FD myopia in guinea pig eyes was effectively blocked by the scleral CXL using sub-Tenon injections of genipin. No histological damage was found on the retina or choroid of these treated eyes. Further studies are needed to examine the long-term efficacy and safety of this CXL technique.

**Electronic supplementary material:**

The online version of this article (doi:10.1186/s12886-015-0086-z) contains supplementary material, which is available to authorized users.

## Background

Myopia, the most prevalent refractive error, affects about 15–38.7 % of the population, and poses a significant public health burden and cost to society [1–3]. The excessive axial eye size, especially the vitreous chamber elongation, is the most important determinant factor of this condition. The longer the axial length, the more severe the myopia [4]. Form deprivation (FD) is an effective approach to induce animal models of myopia. It disrupts the normal growth process, induces rapid axial elongation, and results in myopia in many species [5, 6]. The guinea pig is considered to be a suitable alternative for the mammalian model of FD myopia [7].

Scleral cross-linking (CXL) is a novel attempt to prevent the axial elongation and slow down the myopia process [8]. As a natural CXL reagent derived from *Gardenia jasminoides*, genipin has a potential for neuroprotective action and biocompatibility [9, 10]. Genipin CXL has similar exvivo toxicity in the cornea than in UV-riboflavin CXL, and much less than gluaraldehyde [11]. Scleral CXL using genipin has been proven to be effective in strengthening corneal and scleral tissues [12, 13]. The purpose of this study is to evaluate the effects of scleral cross-linking using genipin in form-deprivation myopia in guinea pigs.

## Methods

### Animals

Twenty-seven 3-week-old pigmented guinea pigs (*Cavia porcellus*) [14] were obtained from the Laboratory Animal Center of Peking University, and were raised on a shift of 12-h illumination and 12-h darkness each day. Water and food were available for the animals. The study adheres to the Association for Research in Vision and Ophthalmology statement for the use of animals in ophthalmology and vision research and was approved by the Ethical Committee of Peking University.

### Form deprivation and scleral cross-linking using genipin

**Fig. 1:**
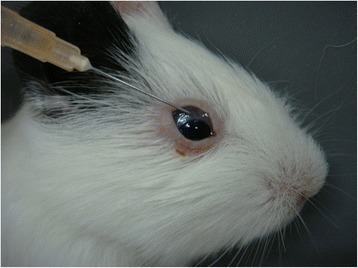
The sub-Tenon injections of 0.50 % genipin solution in guinea pig eyes

The pigmented guinea pigs were randomly divided into three groups (Table 1). Group A (*n* = 8) is the untreated control group which was free from any intervention. Group B (*n* = 8) is the FD group, in which test subjects were covered with a monocular facemask on their right eyes for 21 days [15]. The facemasks were checked every day to ensure that they were in place. All the right eyes in Group C (*n* = 11) were also covered with a monocular facemask to produce FD eyes. In this group, scleral cross-linking using genipin was intervened. A sub-Tenon injection using 0.10 mL of 0.50 % genipin (Wako, Japan) in balanced salt solution (BSS) were performed 3.0 mm behind the limbus in both superonasal and inferotemporal quadrants 3 times (7 days in-between injections) on the FD eyes of Group C (Fig. 1) [16]. All injections were performed under light general anesthesia with 10 % ether in oxygen.

**Table 1:** Treatment protocols for right eyes in the three groups

Groups	Descriptions	FD for 21 days	Sub-Tenon injection with 1.00 % genipin
A (*n* = 8)	Untreated eyes	−	−
B (*n* = 8)	FD eyes	Yes	−
C (*n* = 11)	Eyes with FD and0.5 % genipin CXL	Yes	Yes

*FD* form-deprivation, *CXL* cross-linking

### Biological measurements

Ocular refraction and axial length were measured before and after the induction of the FD process. Cycloplegia was induced with three drops of 1 % tropicamide. Ocular refraction was performed in a dark room with a streak retinoscope and trial lenses. The refraction was calculated as spherical component refractive error (the mean refraction in the horizontal and vertical meridians). A-scan ultrasonography (Aviso, Quantel Medical Inc., France) was used to measure the axial lengths of the eyes. Slit lamp biomicroscopy was performed to check the corneal and lens conditions. All examinations were performed under light general anesthesia with 10 % ether in oxygen.

### Biomechanical measurements

The right eyes of all animals were enucleated after euthanasia with an overdose of pentobarbital sodium. Five eyeballs in Group C were randomly selected and were used for the biomechanical test of the sclera by comparing with Groups A and B. One scleral strip (size, 2.0 mm × 6.0 mm) was dissected from the equatorial sclera of the superonasal quadrant. The scleral thickness was measured using a laser displacement sensor (KEYENCE LK-G30 1-3-14. Higashi-Nakajima, Higashi-Yodogawa-ku, Osaka, Japan). The strips were clamped vertically with a distance of 4.0 mm between the jaws of a microcomputer-controlled biomaterial tester (BOSE Electro Force® Series II 3330; Bose Corp, Bose ElectroForce Systems Group, Eden Prairie, Minnesota, USA). Each specimen was preloaded and preconditioned according to a previously published article [17]. Strain was increased linearly at a velocity of 1 mm/min. The ultimate stress σ (MPa) and ultimate strain ε (%) of the samples were used for biomechanical analysis. Young modulus E (MPa) was calculated for the ultimate strain as the gradient of the stress–strain graph (E = dσ/dε) [18].

### Light microscopy

Three of the remaining eyeballs in Group C (*n* = 3) were randomly selected and fixed in a solution of formalin 10 % for light microscopy; 4-μm-thin paraffin sections were stained with haematoxylin and eosin (H&E).

### Electron microscopy

Other remaining eyeballs in the three groups (each group, *n* =3) were fixed in 2 % glutaraldehyde for transmission electron microscopy (TEM); thin sections (60–90 nm) were stained with lead citrate and uranyl acetate and examined under a transmission electron microscope (JEM-2100, JEOL Ltd., Tokyo, Japan). Electron micrographs (magnification, × 40,000) of collagen fibrils were taken from the scleral middle layer (center bundle) of each eyeball. In total, nine strips measuring 2 μm × 2 μm were separately obtained from nine electron micrographs of three eyeballs in each group. The fibril diameter (nm) and density (/μm^2^) in each group were evaluated using Image-Pro Plus 6.0 (Media Cybernetics, Inc. USA). When fibers were elliptical, the smallest diameter was measured.

### Statistical analysis

Statistical analysis was performed with JMP™ 9 statistical package (SAS Institute, Inc., Cary, NC, USA) software. Descriptive statistical results were presented as mean and standard deviation. Categorical variables were compared using the Pearson’s Chi-Square test. When parametric analysis was possible, One-way Anova with Tukey’s HSD test was used to compare the results among the three groups. When parametric analysis was not possible, the Kruskal-Wallis test withSteel-Dwass test was used to compare the three groups. Results with *P* < 0.05 were considered statistically significant.

## Results

### Biological measurements

No corneal and lens complication was found using slit lamp biomicroscopy at the end of the study. Table 2 showed the differences in axial length and refraction among the three groups at the start (day 0) and end (day 21) of the FD process. In Group B, a significant increase in axial length and refraction was found after the 21-day FD (*P* < 0.05). However, the myopic effects of the FD in Group C were not statistically significant (*P* > 0.05).

**Table 2:** Axial length and refraction of right eyes in the three groups at day 0 and 21 of form deprivation

	Group A (*n* = 8)	Group B (*n* = 8)	Group C (*n* = 11)	*P *Values among 3 groups	*P* values of post hoc Comparison		
					A vs. B	B vs. C	A vs. C
Axial length, mm (day 0)	7.02 ± 0.25	7.14 ± 0.22	7.08 ± 0.21	0.9173			
Axial length, mm (day 21)	10.95 ± 0.54	11.99 ± 0.65	10.73 ± 0.85	0.0026	0.0202	0.0025	NS
Refraction, D (day 0)	3.40 ± 1.01	3.50 ± 0.98	3.75 ± 1.02	0.9334			
Refraction, D (day 21)	2.47 ± 0.95	−2.51 ± 1.15	2.23 ± 1.24	< .0001	< .0001	< .0001	NS

*NS* no statistical significance

### Biomechanical measurements

Table 3 showed the scleral thickness and biomechanical properties of the eyes in the three groups at the end of the current study. Compared with the FD myopia eyes in Group B, the ultimate stress and Young’s modulus of the eyes in Group C were significantly increased (*P* < 0.01).

**Table 3:** Scleral thickness and biomechanical properties of guinea pigs in the three groups

	Group A (*n* = 5)	Group B (*n* = 5)	Group C (*n* = 5)	*P* Values among 3 groups	*P* values of post hoc Comparison		
					A vs. B	B vs. C	A vs. C
Scleral thickness, mm	0.16 ± 0.05	0.13 ± 0.05	0.17 ± 0.04	0.4598			
Ultimate stress, MPa	0.72 ± 0.30	0.46 ± 0.16	0.98 ± 0.19	0.0116	NS	0.0089	NS
Ultimate strain, %	16.74 ± 6.86	21.71 ± 3.58	14.20 ± 3.18	0.0809			
Young modulus, MPa	4.52 ± 1.99	2.17 ± 0.75	7.23 ± 2.36	0.0033	NS	0.0024	NS

*NS* no significance

### Light microscopy

**Fig. 2:**
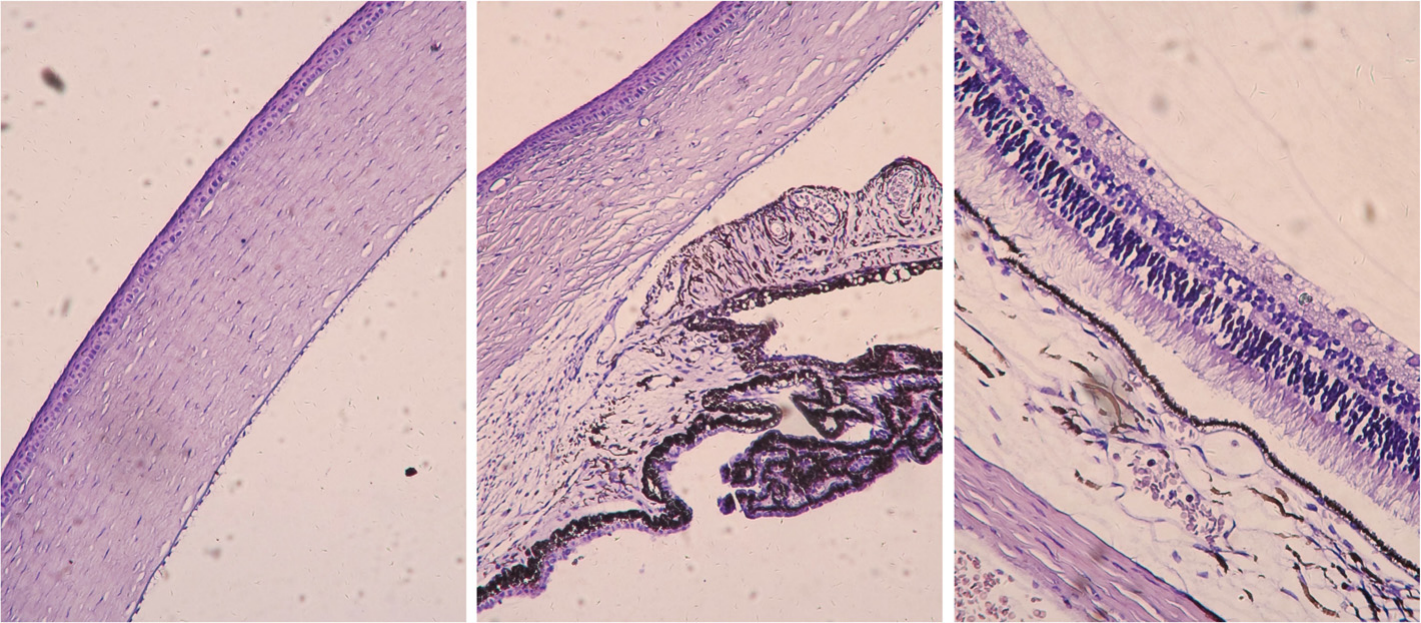
Photomicrograph of guinea pig eye. The photomicrograph of guinea pig cornea (*left*), corneal limbal (*middle*), retina, choroid and sclera (*right*) in a guinea pig eye after the form deprivation and genipin CXL are shown (H&E stain; original magnification × 200)

Mild edema was seen in the peripheral cornea and sclera adjacent to the injection site in the eyes of Group C (Fig. 2, left and center). The endothelium was intact. The retina and choroid were observed without abnormalities (Fig. 2, right).

### Electron microscopy

**Fig. 3:**
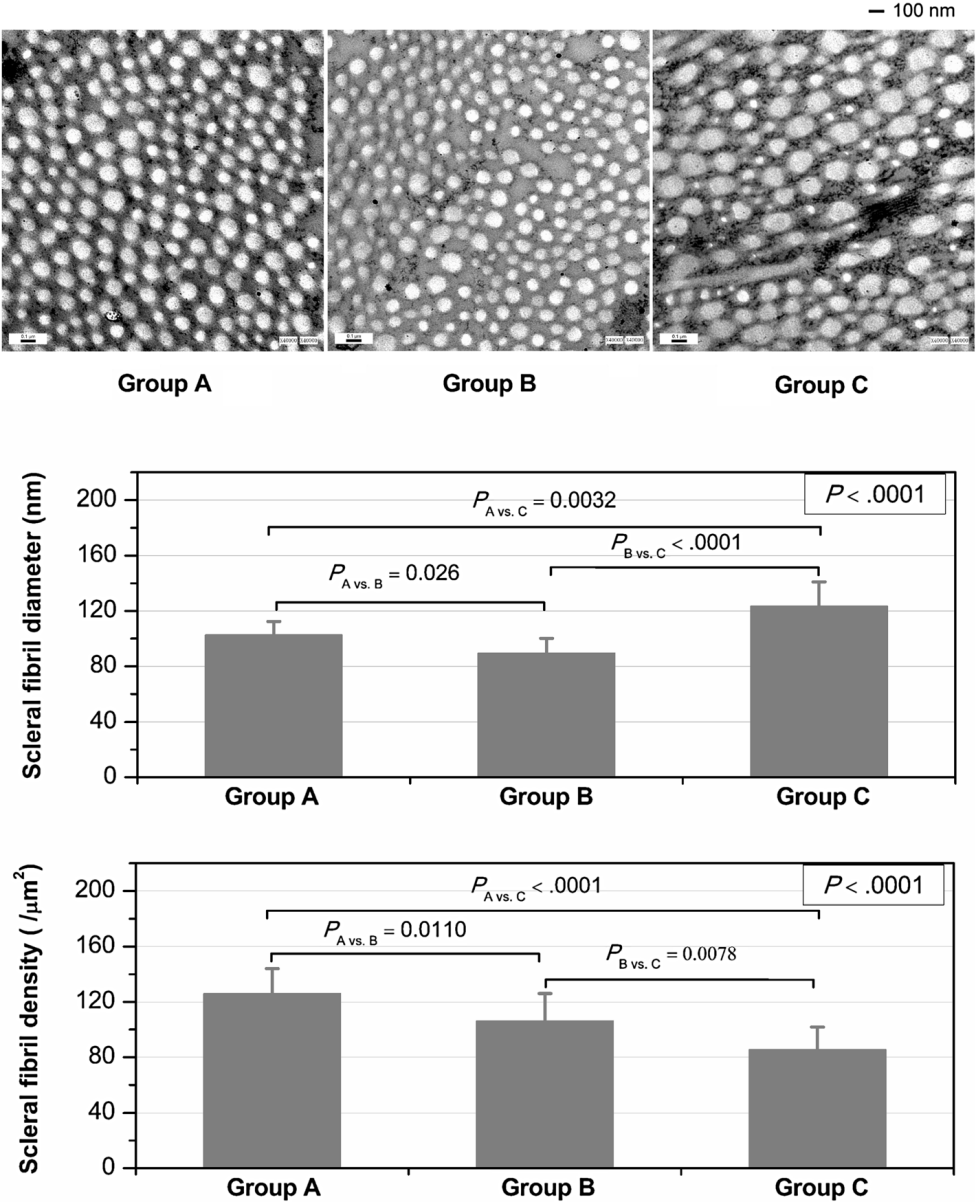
Electron micrographs of guinea pig sclera. Upper figure: Electron micrographs (original magnification × 40,000) showing the scleral collagen fibrils of untreated eyes(*Group A*); eyes after the 21-day form deprivation FD (*Group B*); and eyes after both form deprivation and scleral CXL (*Group C*). Middle figure: The scleral fibril diameters of guinea pig eyes in the three groups. Lower figure: The scleral fibril density of guinea pig eyes in three groups

There were statistically significant differences in the diameter and density of the scleral fibrils among the three groups (*P* < .0001, Fig. 3). Compared with the untreated control eyes in Group A (fibril diameter, 102.56 ± 9.82 nm; fibril density, 126.00 ± 13.13 /μm^2^), both the scleral fibril diameter (89.44 ± 10.72 nm) and density (106.11 ± 19.99 /μm^2^) of eyes after 21-day FD (Group B) were statistically decreased (*P* = 0.0026 and *P* = 0.0110, respectively). After the 21-day FD and genipin CXL, the fibril diameter in Group C reached to 123.44 ± 17.55 nm, which was statistically larger than the untreated control (*P* = 0.0032) and FD control (*P* < .0001) eyes; on the other hand, the fibril density (85.33) in Group C reduced to 85.33 ± 16.52 /μm^2^, which was statistically less than the two control groups (*P* < 0.01).

## Discussion

Form deprivation is an effective technique to establish the myopia model of the guinea pig eye [7]. Significant amounts of myopia, axial elongation and thinning of the scleral fibril diameter can be found in the guinea pig eyes after 21-day FD [14]. In the present study, the average axial length of the FD eyes in Group B (11.99 mm) was 109 % of the untreated control eyes in Group A (10.95 mm); −6 D of myopia (from 3.50 to −2.51 D) was developed in the guinea pig eyes at the end of this FD process. Although changes induced by FD were observed throughout the whole eye after FD, the elongation of the vitreous chamber and the thinning of the sclera were the dominant responses [7, 14]. These ocular changes were similar to the myopic process in human and monkey eyes [19, 20].

Cross-linking plays an important role for the regulation of ocular elongation and myopia development [21]. Intraperitoneal injections of cross-linking blocker (such as β-aminoproprionitrile and D-penicillamine) have been found to increase the FD myopia of tree shrew eyes [21]. As a physical CXL technique, scleral CXL using ultraviolet A (UVA) and riboflavin has been proven to strengthen the scleral biomechanical rigidity without any obvious side-effects [22]. Compared with the clearly defined target area of physical CXL technique, sub-Tenon injections with chemical CXL reagents have several advantages, such as minimally invasive procedure (via small incision), large treatment area (may include the entire sclera), and ease of manipulation without the need for any specialized UV device nor UV irradiation [16].

The amount of axial myopia was found to correlate with the mechanical properties of the sclera [23]. As a natural CXL reagent, genipin has remarkable efficacy [24], stability [25] and low cytotoxicity [10], and has been used for improving the mechanical property of scleral tissue [12]. In previous ex vivo studies [13, 26], it has been found that the stiffness of porcine sclera increases 280–820 % after a 30-min incubation with 1 % genipin. In the present study, the scleral Young modulus in the eyes with FD and genipin CXL (Group C, 7.23 MPa) was similar to that in the untreated control eyes (Group A, 4.52 MPa; *P* = 0.0873), and was 3 times stronger than those eyes with FD only (Group B, 2.17 MPa; *P* = 0.0024). On the other hand, sub-Tenon injections using 0.50 % genipin eliminated the eye elongation and stopped the myopia development induced by the FD in guinea pig eyes. At the end of this study, the amount of axial length and myopia were statistically less than the FD eyes in Group B (*P* < 0.01), and were statistically equal to the value of the untreated eyes in Group A (*P* > 0.05).

Furthermore, the development of FD myopia in mammalian eyes is associated with the ultrastructural performances of scleral fibrils [27]. It has been proven that smaller diameter collagen fibrils provide tissues with lower tensile strength [28]. In this study, the FD induced the axial elongation and myopic growth in guinea pig eyes, which was accompanied with a decrease in scleral fibril diameter; in turn, the genipin CXL eliminated the FD-induced axial elongation and prevented the myopic development accompanied with the thickening of scleral collagen fibrils. However, unlike the adenosine receptor antagonists (such as 7-methylxanthine) [14], the thickening of the target sclera was not present in the current and in some previous studies [17]. This is because CXL is a process of bonding the present existing collagen fibrils, but not a process of collagen regeneration. In the present study, both the increase in fibril diameter and the reduction of fibril density were observed under the transmission electron microscope. Previous studies also found the damages of fibroblasts and the thinning of corneal or scleral tissues after CXL procedures [29]. Long-term efficacy of the scleral CXL using genipin for halting the process of FD myopia should be observed in the future.

In the present study, mild reversible side effects were observed in the peripheral cornea and sclera adjacent to the injection sites, whereas the retina and choroid were not affected. The ocular barriers were believed to play important roles in protecting the retina and choroid from the histological damages of the sub-Tenon injection and genipin toxicity [16]. Yet, it was observed that genipin toxicity is dose-dependent but not time-dependent [13]. The promising genipin concentration and treatment time in tissue-engineering practices are 0.5 mM (equivalent to 0.0113 %) and 30 min, respectively [13]. This is much lower than the present dose used in our study. Thus, more attention should be concentrated on the visual function of cross-linked eyes in the future to examine the possible cytotoxic effects of this technique.

In addition, some limitations of the current study should be noted. The limited number of eyes, as well as the time-frame, for instance, cannot elaborate information about the long-term efficacy and safety of the current CXL technique. Additionally, the changes in intraocular pressure and optic nerve were not investigated in the current study.

## Conclusions

In conclusion, the process of FD myopia in guinea pig eyes was effectively blocked after the scleral CXL technique using sub-Tenon injections of genipin. No histological damage was found in the retina or choroid of these cross-linked eyes. Further studies using more animal eyes or donor human eyes are needed to examine the long-term efficacy and safety of this CXL technique.

## Additional file

Additional file 1.
**The ARRIVE Guidelines Checklist.** (PDF 1067 kb)
